# Trends and factors associated with teenage pregnancy in Ethiopia: multivariate decomposition analysis

**DOI:** 10.1038/s41598-024-52665-5

**Published:** 2024-01-26

**Authors:** Melkamu Aderajew Zemene, Fentaw Teshome Dagnaw, Denekew Tenaw Anley, Enyew Dagnew, Amare Zewdie, Aysheshim Belaineh Haimanot, Anteneh Mengist Dessie

**Affiliations:** 1 Department of Public Health, College of Health Sciences, Debre Tabor University, Debre Tabor, Ethiopia; 2 Department of Midwifery, College of Health Sciences, Debre Tabor University, Debre Tabor, Ethiopia; 3 Department of Public Health, College of Medicine and Health Sciences, Wolkite University, Wolkite, Ethiopia; 4 Department of Public Health, College of Health Sciences, Debre Markos University, Debre Markos, Ethiopia

**Keywords:** Diseases, Health care, Medical research, Risk factors

## Abstract

Teenage is a time of transition from childhood to adulthood. This stage is a time of change and needs particular care and ongoing support. Adolescent pregnancy remains a common health care problem in low- and middle-income countries, and it is associated with higher maternal and neonatal complications. Thus, this study aimed to determine the trends and factors associated with them that either positively or negatively contributed to the change in teenage pregnancy in Ethiopia. Ethiopian Demographic and Health Survey data from 2005 to 2016 were used for this study. A total weighted sample of 10,655 (3265 in 2005, 4009 in 2011, and 3381 in 2016) teenagers was included. Trends and the proportion of teenage pregnancies for each factor over time were explored. Then, a logit-based multivariate decomposition analysis for a non-linear response model was fitted to identify the factors that contributed to the change in teenage pregnancy. Statistical significance was declared at p-value < 0.05 and the analysis was carried out on weighted data. Teenage pregnancy declined significantly from 16.6% (95% CI: 15.4, 17.9) to 12.5% (95% CI: 11.4, 13.6) in the study period, with an annual reduction rate of 2.5%. About 49.8% of the decrease in teenage pregnancy was attributed to the change in the effect of the characteristics. The compositional change in primary educational status (41.8%), secondary or above educational status (24.55%), being from households with a rich wealth index (1.41%) were factors positively contributed to the decline in teenage pregnancy, whereas being from a Muslim religion (−12.5%) was the factor that negatively contributed to the reduction in teenage pregnancy. This study has shown that teenage pregnancy declined significantly; however, it is still unacceptably high. The changes in compositional factors of teenagers were responsible for the observed reduction in the prevalence of teen pregnancy rates in Ethiopia. Educational status, religion, and wealth index were found to be significant factors that contributed to the reduction in teenage pregnancy. Therefore, intervention programs targeting adolescents should address the socio-economic inequalities of these influential factors to reduce teenage pregnancy and related complications.

## Introduction

Teenage is a time of transition from childhood to adulthood. World Health Organization (WHO) defines the age group 10–19 years as the adolescent stage and teenagers are from 13 to 19 years adolescents^1^. This stage is a time of change and needs particular care and ongoing support. Their life is in grave danger due to physical, emotional, mental, and social changes^2^. Teenage childbearing among adolescents aged 15 to 19 is a common sexual and reproductive health (SRH) issue among young people, particularly in developing countries. It is associated with higher maternal and neonatal complications^3^. When a girl between the ages of 15 and 19 becomes pregnant for the first time or gives birth, it is referred to as teenage pregnancy or adolescent childbearing. Teenage pregnancy may drastically alter the girl’s life. For instance, teenage pregnancy often disrupts a teenager’s education, with many drooping out of school or experiencing difficulties in completing their education. This can limit their future opportunities for employment and economic stability. As a result, teenage pregnancy creates a group of young ladies with little education and little economic options who are unable to contribute to the country's progress^4^.

Every year, 2 million teenagers under the age of 15 and around 16 million adolescent girls between the ages of 15 and 19 give birth. Nearly 95% of these births which make up around 11% of all births worldwide take place in underdeveloped nations. Teenage pregnancy rates varied by region, from 2% in China to 18% in Latin America and the Caribbean to more than 50% in sub-Saharan Africa^5^.

Countries in SSA continue to have high rates of teenage pregnancies, despite many policies and programs are designed to lower this number^6^. Evidence from systematic review and meta-analysis showed that the pooled prevalence of adolescent pregnancy in Africa was 18.8%, and it was 19.3% in sub-Saharan Africa region. The highest prevalence was in East Africa with 21.5%, and the lowest was from North Africa with 9.2%^7^. Moreover, teenage pregnancy among adolescents who had ever had sex ranged from 36.5% in Rwanda to 75.6% in Chad^8^. In Zambia, teenage pregnancy has shown an overall decrease of only 2% in a period more than a decade and half^9^. In Uganda, teenagers contributed substantially to live births (26.7%), stillbirths (19.2%), low birth weight infants (42.7%), and referrals (17.3%)^10^. In Tanzania teenage pregnancy increased from the year 2010 to 2016^4^. The prevalence rate of teenage pregnancies seems to be on the increase, especially in rural communities of Zimbabwe^11^.

Findings from systematic review and meta-analysis reported the prevalence of teenage pregnancy in Ethiopia ranges from 12.5 to 30.2%^12^. Evidence from national studies revealed that teenage pregnancy reduced from 16.3% in 2000 to 12.5% in 2016^13^. On the other hand, a local study among school adolescents of Arba Minch Town, Southern Ethiopia revealed a 7.7% teenage pregnancy^14^. Another study in Kersa District, East Haraghe Zone, Oromia Regional State reported a high prevalence of teenage pregnancy with 30.2%^15^.

Numerous factors contribute to sub-Saharan Africa's high rates of adolescent pregnancies. The three main themes identified by studies as impacting adolescent pregnancies are social and economic, personal, and health service-related variables. Adolescent pregnancy rates may be decreased by community awareness, thorough sexual health education, and ensuring women education^7,16,17^.

Age, contraceptive utilization, marital status, working status, household wealth status, community-level contraceptive utilization, age at initiation of sex, media exposure, educational level, and relation to the household head were associated with adolescent pregnancy^12,17–19^. Moreover, studies in Ethiopia showed that sexual practice before the age of 15 years, not being in school, parental divorce, having an elder sister who had a history of teenage pregnancy, and not knowing fertile period in the menstrual cycle were factors associated with teenage pregnancy^3,12,18,20,21^.

Even though there have been different local studies on the prevalence and factors associated with teenage pregnancy in Ethiopia, to the best knowledge of the authors, there is limited evidence on the factors either positively or negatively contributed to the change in teenage pregnancy so far in Ethiopia. Therefore, this study aimed to examine trends, and the factors that contributed to the change in the prevalence of teenage pregnancy in Ethiopia. Thus, findings from this study will help policymakers, program managers, and scholars in evaluating and designing strategies targeting influential factors to reduce teenage pregnancy. Moreover, the results of this study will be crucial for developing intervention programs to reduce teenage childbearing and related complications.

## Methods and materials

### Data source

Secondary data analysis was conducted based on the Ethiopian Demographic and Health Survey (EDHS) of 2005, 2011, and 2016. EDHS is a nationally representative population-based survey that has been conducted every five years. Demographic and Health Survey used a two-stage stratified cluster sampling technique. In the first stage, a sample of EAs was selected independently from each stratum with proportional allocation stratified by residence. In the second stage, from the selected EAs, households were taken by systematic sampling technique. The data were accessed from the DHS program official database https://www.measuredhs.com after permission was granted through an online request.

### Study population

The source population was all teenagers in five years preceding each respective survey in Ethiopia, whereas those in the selected Enumeration Areas (EAs) were the study population. The sample size was determined from the individual to recode file “IR file”. A total weighted sample of 10,655 (3265 in 2005, 4009 in 2011, and 3381 in 2016) teenagers was included in this study.

### Study variables

#### Outcome variable

The outcome variable was teenage pregnancy taken as a binary response; 0 coded for “no” and 1 coded for “yes”.

#### Independent variables

The independent variables include; educational status (no education, primary education, and secondary or above), family size (less than six, greater than or equal to six), residence (urban, rural), religion (Orthodox, Muslim, Protestant, others), occupation (employed, not employed), marital status (“unmarried” which includes never in union, separated, divorced, widowed, and “married”), husband education (no education, primary education, and secondary or above), sex of head of the household (male, female), media exposure (yes, no), wealth index (poor, middle, rich), age at first marriage, contraceptive use (using modern methods, using traditional methods, non-user-intend to use later, and do not intend to use), age at first intercourse (never had sex, active before the age of 18, and active after 18 years), and age at first birth (gave birth before 18 years, gave birth after the age of 18 years) (Table 1).

**Table 1:** Description and measurement of independent variables.

Variables	Description/categorization
Place of residence	The variable residence was recoded as rural and urban without any change in the dataset
Age	Age of the adolescent as a continuous variable
Media exposure	A composite variable obtained by combining whether the adolescent girls listen to radio, read magazine/newspaper, and watch television. It was recoded as “0” if the adolescent girls were not exposed to at least one of the three medias, and “1” if they had exposure to at least one of the three medias
Educational status	This is the highest educational level during interview an adolescent girl achieved and recoded into “0” for no education, “1” for primary education, and “2” for secondary or above
Marital status	Refers the current marital status of the adolescent girl and recoded into two categories as “0” for unmarried (includes never in union, separated, divorced, and widowed), and “1” for married (includes those were married and living with partner)
Sex of household head	The variable sex of household head was recoded as male and female in the dataset and used as it is
Religion	The variable religion was recategorized as “0” for orthodox, “1” for Muslim, “2” for protestant, and “3” for others (catholic, traditional, others)
Occupation	Recoded into “0” for not employed, and “1” for employed
Wealth index	Wealth index was created using principal component analysis and coded as “poorest”, “poorer”, “middle”, “richer”, and “richest” in EDHS dataset. In this study, we recategorized into three groups as poor (includes poorer and poorest), middle, and rich (includes richer and richest)
Age at first sex	Recoded as “0” for never had sex, “1” for active before the age of 18, and “2” for active after 18 years
Age at first birth	Recoded as “yes” for those who gave birth before 18 years, and “no” for “no” for who gave birth after the age of 18
Age at first marriage (cohabitation)	Refers the age of the adolescent girls at first marriage and used as a continuous variable
Contraceptive use and intension to use	This variable is recoded as “0” for using modern methods, “1” for using traditional methods, “2” for non-user-intend to use later, and “3” for do not intend to use
Drinking water source	Recoded as “1” for households who were getting drinking water from improved water sources (includes piped water sources, protected spring, protected well, and rainwater), and “0” for else
Type of toilet facility	Recoded as “1” for households who had improved toilet facility (includes flush to piped sewer system, flush to septic tank, flush to pit latrine, ventilated improved pit latrine, and composting toilet), and “0” for else

### Operational definition

#### Teenage pregnancy

Teenage pregnancy is the percentage of adolescent girls who have begun childbearing, that is the sum of the percentage who gave birth and/ or the percentage who are pregnant with their first child^22^.

#### Wealth index

Wealth index is a composite measure of a household’s cumulative living standard divided into five quantiles using the wealth quantile data derived from the principal component analysis^22^. In this study, we recategorized into three groups as poor (includes poorer and poorest), middle, and rich (includes richer and richest).

### Statistical analysis

The data were analyzed by using Stata version 16/MP software. All statistical analysis was conducted on weighted data to account for DHS complex survey design. First, a descriptive analysis was done to examine the trends with a 95% confidence interval (CI) of teenage pregnancy. Similarly, the proportion of teenage pregnancy by study participants’ characteristics was explored. Then, a logit-based multivariate decomposition analysis for a non-linear response model was implemented to determine the extent to which factors contributed to the observed change in teenage pregnancy.

In the bivariate analysis, variables with a p-value of less than 0.25 were selected for multivariate decomposition analysis. Then, a p-value of less than 0.05 with 95% CI was used to declare statistical significance after fitting to multivariate decomposition analysis in the overall decomposition.

### The multivariate decomposition analysis model

Multivariate decomposition analysis for a non-linear model is used to split the difference in a distribution statistic between two groups, or its change over time, into various explanatory factors. This statistical approach uses the output from regression models to partition the components of a group difference in a statistic, such as a mean or proportion, into a component attributable to compositional differences between groups; differences in characteristics (endowments), and a component attributable to differences in the effects of characteristics (differences in coefficients). This analysis technique is equally applicable for partitioning change over time into components attributable to changing composition and changing effects^23–26^.

The dependent variable is the function of the linear combination of predictors and regression coefficients.

$${\text{Y}}\, = \,{\text{F}}({\text{X}}\beta )$$

where Y denotes the N × 1 dependent variable vector, X is an N × K matrix of independent variables, and β is a K × 1 vector of coefficients. F (·) is any once-differentiable function mapping a linear combination of X(Xβ) to Y. The overall differences in components that reflect compositional differences between groups (endowments) and differences in the effects of characteristics (coefficients) between two groups A and B can be decomposed as:

$${\text{YA}}\, - \,{\text{YB}}\, = \,{\text{F}}\left( {{\text{XA}}\beta {\text{A}}} \right)\, - \,{\text{F}}({\text{XB}}\beta {\text{B}})$$

$$\begin{gathered} {\text{Logit }}\left( {{\text{Y}}_{{\text{A}}} } \right){-}{\text{logit }}\left( {{\text{Y}}_{{\text{B}}} } \right)\, = \,{\text{F }}\left( {{\text{X}}_{{\text{A}}} \beta_{{\text{A}}} } \right){-}{\text{F }}({\text{x}}_{{\text{B}}} \beta_{{\text{B}}} ) \hfill \\ \, = \,\underbrace {{{\text{F }}\left( {{\text{X}}_{{\text{A}}} \beta_{{\text{A}}} } \right){-}{\text{F }}\left( {{\text{X}}_{{\text{B}}} \beta_{{\text{A}}} } \right)}}_{{\text{E}}}\, + \,\underbrace {{{\text{F }}\left( {{\text{X}}_{{\text{B}}} \beta_{{\text{A}}} } \right){-}{\text{F }}({\text{X}}_{{\text{B}}} \beta_{{\text{B}}} )}}_{{\text{C}}} \hfill \\ \end{gathered}$$

The component labeled “E” refers to the part of the differential attributable to differences in endowments or characteristics, usually called the explained component or characteristics effect. The “C” component is the difference attributable to coefficients (behavioral change) usually called the unexplained component. We have chosen group A as the comparison group and group B as the reference group. Thus, E reflects a counterfactual comparison of the difference in outcome from group A’s perspective (i.e., the expected difference if group A were given group B’s distribution of covariates). C reflects the counterfactual comparison of the difference in outcome from group B’s perspective (i.e., the expected differences if group B were experienced in group A’s behavioral response to X).

In this study, we applied a decomposition analysis to account for changes in teenage pregnancy between 2005 and 2016. The model for decomposition analysis was: Logit (A) − Logit (B) = [β0A − β0B] + ΣβijA [XijA − XijB] + ΣxijB[βijA − βijB]^23^

Where,

- β0A was the intercept in the regression equation for EDHS 2016.
- β0B was the intercept in the regression equation for EDHS 2005.
- βijA was the coefficient of the jth category of the ith determinant for EDHS 2016.
- βijB was the coefficient of the jth category of the ith determinant for EDHS 2005
- XijA was the proportion of the jth category of the ith determinant for EDHS 2016.
- XijB was the proportion of the jth category of the ith determinant for EDHS 2005.

### Ethical approval

Permission to access the data was obtained from the webpage of the International Review Board of Demographic and Health Survey (DHS) program. The dataset is publicly available in requesting a concept note for a proposed project from (https://www.measuredhs.com). Initially, the Ethiopian Demographic and Health Survey (EDHS) followed its ethical procedures and the detail is also available in the full report^22^.

## Results

### Socio-demographic characteristics

In this study, a total weighted sample of 10,655 adolescent teens were included. The mean ± Standard Deviation (SD) of age was almost similar with 16.9 ± 1.3 years. Regarding educational status, adolescent girls who were not educated decreased from 40% in 2005 to 13.8% in 2016. The proportion of teenagers who had media exposure increased from 42.5 to 52.4% in the same period (Table 2).

**Table 2:** Socio-demographic characteristics of teens in Ethiopia using the 2005–2016 EDHS.

Variables	Categories	2005 EDHS, n (%)	2011 EDHS, n (%)	2016 EDHS, n (%)
Age	Mean ± SD	16.9 ± 1.37	16.8 ± 1.39	16.9 ± 1.3
Age distribution	15	729 (22.3)	1006 (25.1)	708 (20.9)
	16	667 (20.4)	821 (20.5)	701 (20.7)
	17	556 (17.0)	627 (15.6)	642 (18.9)
	18	862 (26.4)	977 (24.4)	913 (27.0)
	19	451 (13.8)	578 (14.4)	417 (12.3)
Educational status	No education	1308 (40)	695 (17.3)	468 (13.8)
	Primary	1423 (43.6)	2813 (70.2)	2147 (63.6)
	Secondary & above	535 (16.4)	501 (12.5)	765 (22.6)
Family size	< 6	1442 (44.2)	1768 (44.1)	1550 (45.8)
	≥ 6	1823 (55.8)	2241 (55.9)	1831 (54.2)
Religion	Orthodox	1703 (52.2)	2013 (50.4)	1426 (42.2)
	Muslim	859 (26.3)	1075 (26.8)	1064 (31.2)
	Protestant	606 (18.6)	833 (20.8)	847 (25.0)
	Others*	97 (2.9)	78 (2)	44 (1.3)
Residence	Urban	703 (21.5)	1042 (26)	804 (23.8)
	Rural	2562 (78.5)	2967 (74)	2576 (76.2)
Occupation	Not working	2346 (71.8)	2038 (50.8)	1994 (59)
	Working	919 (28.2)	1971 (49.2)	1386 (41)
Marital status	Unmarried	2394 (73.3)	3087 (77)	2641 (78.1)
	Married	871 (26.7)	922 (23)	739 ()21.9
Husband education	No education	519 (59.6)	417 (45.3)	205 (34.9)
	Primary	273 (31.3)	400 (43.4)	266 (45.3)
	Secondary or above	78 (9.1)	104 (11.3)	116 (19.8)
Drinking water source	Improved	1776 (54.4)	2012 (50.2)	1814 (53.7)
	Unimproved	1489 (45.6)	1997 (49.8)	1566 (46.3)
Toilet facility	Improved	414 (12.7)	741 (18.5)	579 (17.1)
	Unimproved	2851 (87.3)	3268 (81.5)	2801 (82.9)
Sex of head of HH	Male	2499 (76.5)	2922 (72.9)	2499 (73.9)
	Female	766 (23.5)	1087 (27.1)	882 (26.1)
Media exposure	Yes	1389 (42.5)	3071 (76.6)	1773 (52.4)
	No	1876 (57.5)	937 (23.4)	1608 (47.6)
Wealth index	Poor	1012 (31)	1382 (34.4)	1036 (30.6)
	Middle	623 (19.2)	687 (17.1)	637 (18.8)
	Rich	1625 (49.7)	1946 (48.4)	1707 (50.5)
Age at first marriage(cohabitation)	Mean ± SD	14.6 ± 2.2	15.2 ± 2.0	15.4 ± 1.6
Early sexual intercourse (before 18 years)	Yes	841 (25.7)	935 (23.3)	741 (22)
	No	62 (2)	35 (0.88)	91 (2.6)
	Never had sex	2361 (72.3)	3038 (75.8)	2549 (75.4)
Knowledge of any contraceptive	Knows no method/traditional	617 (18.91)	179 (4.48)	105 (3.11)
	Knows modern method	2649 (81.2)	3830 (95.5)	3275 (96.9)
Contraception use & intention	Using contraception	83 (2.6)	214 (5.4)	254 (7.5)
	Does not intend to use	3182 (97.4)	3795 (94.6)	3127 (92.5)
Fertility preference	Have another pregnancy	2303 (70.5)	3225 (82.9)	2503 (74)
	Undecided	170 (5.2)	207 (5.2)	632 (18.7)
	No more	728 (22.9)	471 (11.7)	245 (7.2)
Gave birth before 18 years	Yes	103 (3.2)	81 (2.1)	102 (3.1)
	No	3162 (96.8)	3928 (97.9)	3278 (96.9)

*HH* household.

*Catholic/traditional/other.

### Sexual and reproductive characteristics

The mean ± SD of age at first marriage was 14.6 ± 2.2 in EDHS 2005 which raised to 15.4 ± 1.6 in EDHS 2016. About one-fourth (25.7%) of adolescent girls were sexually active before their eighteenth birth date in 2005 which later slightly decreased to 22% in 2016. Regarding knowledge of contraceptives, around 18.9% of them did not know any contraceptive methods in 2005 but decreased to 3% in 2016 (Table 2).

### Trend of teenage pregnancy

**Figure 1:**
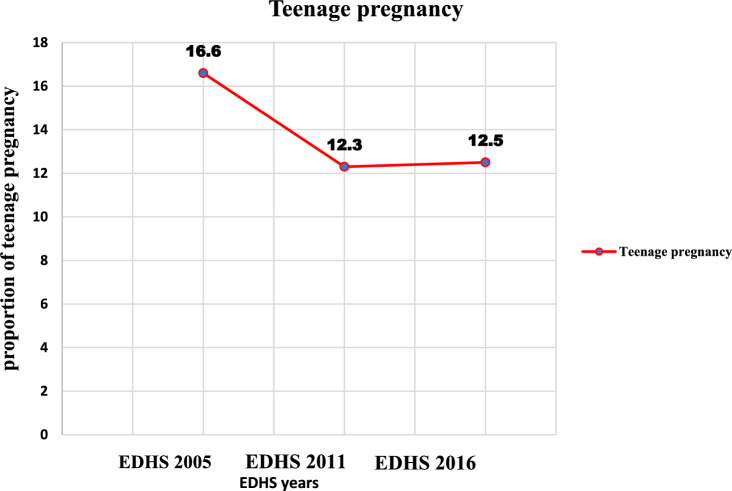
Trend of teenage pregnancy in Ethiopia by using data from EDHS 2005–2016.

Overall, the trend of teenage pregnancy decreased significantly (p < 0.001) with a 4.1% percent point change from 16.6% (95% CI: 15.4, 17.9) to 12.5% (95% CI: 11.4, 13.6) in the study period with an annual reduction rate of 2.5% (Fig. 1). In the first phase (2005–2011), teenage pregnancy reduced from 16.6% (95% CI: 15.4, 17.9) to 12.3% (95% CI: 11.3, 13.4) (Fig. 1).

The trend of teenage pregnancy over the study period (2005–2016) varied in terms of different factors. For instance, the overall change in teenage pregnancy among rural residents was 4.6% in the study period. Based on region, the highest reduction was observed in Gambella (14.6%) percentage change followed by Beneshangul-gumz (13.5%) region. The proportion of teenage pregnancy among the orthodox religion decreased from 15.8% in 2005 to 7.7% in 2016. The prevalence of teenage pregnancy was found high among adolescents who had no media exposure 22.6% as compared to 12.2% of those who had media exposure in 2005. And it decreased to 16.7% and 8.7% in 2016 respectively (Table 3).

**Table 3:** Trends of teenage pregnancy by selected background characteristics in Ethiopia from 2005 to 2016 EDHS in percent (%).

Variables	Categories	EDHS 2005, n = 3265	EDHS 2011, n = 4009	EDHS 2016, n = 3381	Point difference rate in teenage pregnancy		
					2005–2011	2011–2016	2005–2016
Residence	Urban	6.6	4.1	4.9	2.5	−0.8	1.7
	Rural	19.4	15.3	14.8	4.1	0.5	4.6
Region	Tigray	14.7	11.9	11.9	2.8	0	2.8
	Afar	20.3	15.1	23.4	5.2	−8.3	−3.1
	Amhara	20.3	11.6	8.3	8.7	3.3	12
	Oromia	18.9	15.8	16.9	3.1	−1.1	2
	Somali	19.5	19.2	18.7	0.3	0.5	0.8
	Ben-gumz	27.1	19.3	13.6	7.8	5.7	13.5
	SNNP	11	8.2	10.6	2.8	−2.4	0.4
	Gambella	30.8	20.5	16.2	10.3	4.3	14.6
	Harari	21.9	14.5	16.9	7.4	−2.4	5
	Addis Ababa	4.2	2.6	3.0	1.6	−0.4	1.2
	Dire Dawa	13.7	7.6	12.5	6.1	−4.9	1.2
Educational status	No education	28.9	32.8	27.8	−3.9	5	1.1
	Primary	10.4	8.8	12.1	1.6	−3.3	−1.7
	Secondary or above	2.9	4.2	4.0	−1.3	0.2	−1.1
Religion	Orthodox	15.8	11.4	7.7	4.4	3.7	8.1
	Muslim	22.9	14.5	20.5	8.4	−6	2.4
	Protestant	11.1	11.7	10.4	−0.6	1.3	0.7
	Others*	9.3	15.0	15.5	−5.7	−0.5	−6.2
Occupation	Not working	18.2	13.6	13.1	4.6	0.5	5.1
	Working	12.5	11.1	11.6	1.4	−0.5	0.9
Media exposure	Yes	12.2	10.3	8.7	1.9	1.6	3.5
	No	22.6	19.0	16.7	3.6	2.3	5.9
Early sexual intercourse	Active before 18	61.0	52.1	54.9	8.9	−2.8	6.1
	Active after 18	47.5	22.4	16.2	25.1	6.2	31.3
Wealth index	Poor	44.6	34.4	41	10.2	−6.6	3.6
	Middle	19.8	17.2	14.9	2.6	2.3	4.9
	Rich	26.5	14.7	13.9	11.8	0.8	12.6
Overall		16.6 (15.4, 17.9)	12.3 (11.3, 13.4)	12.5 (11.4, 13.6)	4.3	−0.2	4.1

### Factors associated with a change in teenage pregnancy

#### Decomposition analysis

There has been a significant decline in the overall change in teenage pregnancy in Ethiopia from 2005 to 2016. The overall decomposition revealed that the decline in teenage pregnancy over time was explained by the difference in characteristics (endowments). However, the change due to the difference in the effect of the selected explanatory variables was not found to be significant (Table 4).

**Table 4:** Overall decomposition of teenage pregnancy in Ethiopia, 2005–2016.

Teenage pregnancy	Coefficient	(95% CI)	Percent
E	−0.020687	−0.039383, −0.0019903	49.8*
C	−0.020844	−0.051477, 0.0097887	50.2
R	−0.041531	−0.064504, −0.018558	

*E* endowment, *C* coefficient, *R* residual.

*Significant at p < 0.05.

After controlling the role of change in coefficients, 49.8% of the decline in teenage pregnancy in Ethiopia was due to differences in characteristics (endowments). Thus, educational status, religion, and wealth index had a statistically significant contribution to the change in teenage pregnancy. Keeping the other variables constant, as the result of an increase in the proportion of adolescents in primary, secondary, and above school in the survey years (Table 1) had a statistically significant positive contribution to the decline of teenage pregnancy (Table 5). Followers of the Muslim religion were more likely to get married and became pregnant earlier. As a result, an increase in the proportion of Muslim followers in the survey (Table 1) had a significant negative impact on the decline in teenage pregnancy (Table 5). Regarding the wealth index, the change in the composition of the survey population from households with the rich wealth index resulted in a significant positive impact on the decrement of teenage pregnancy (Table 5).

**Table 5:** The detail decomposition analysis of teenage pregnancy in Ethiopia, 2005–2016.

Variables	Differences due to characteristics(E)		Differences due to coefficients (C)	
	Coeff (95% CI)	Pct (%)	Coeff (95% CI)	Pct (%)
Residence				
Urban	Ref	Ref	Ref	Ref
Rural	−0.00075(−0.00235, 0.00084)	1.8174	−0.05496(0.29188, 0.18194)	132.35
Media exposure				
No	Ref	Ref	Ref	Ref
Yes	0.00040 (−0.00199, 0.00281)	−0.97543	−0.01475 (0.10408, 0.07456)	35.528
Educational status				
No education	Ref	Ref	Ref	Ref
Primary	−0.01737(−0.03056, −0.00417)*	41.825	0.06142 (−0.10989, 0.23273)	−147.89
Secondary or above	−0.01019 (−0.01567, −0.00472)***	24.55	0.04885 (−0.09328, 0.1910)	−117.65
Sex of head of HH				
Male	Ref	Ref	Ref	Ref
Female	−0.00107 (−0.00231, 0.00015)	2.5932	0.03271 (0.069925, 0.13536)	−78.776
Religion				
Orthodox	Ref	Ref	Ref	Ref
Muslim	0.00520 (0.00258, 0.00782)***	−12.529	0.05024 (0.077667, 0.17817)	−120.9
Protestant	0.00297 (−0.00080, 0.00676)	−7.1748	0.05301 (0.087929, 0.19396)	−127.6
Others^#^	−0.00035 (−0.00362, 0.00290)	0.86498	0.01149 (0.023785, 0.04678)	
Occupation				
Not-employed	Ref	Ref	Ref	Ref
Employed	0.00099 (−0.00433, 0.00632)	−2.3882	0.05066 (0.086328, 0.18766)	−121.9
Wealth index				
Poor	Ref	Ref	Ref	Ref
Middle	0.00007 (−0.00010, 0.00025)	−0.18214	−0.01299 (0.05892, 0.03294)	31.277
Rich	−0.00058 (−0.00097, −0.00019)**	1.4086	−0.09715 (0.35026, 0.15595 )	233.94
Overall	−0.02068 (−0.03938, 0.00199)*	49.8	−0.020844 (0.051477, 0.0097887)	50.2

^#^Catholic/traditional/other.

*Significant at p < 0.05; **significant at p < 0.01; ***significant at p < 0.001.

*HH* household, *Ref* reference, *CI* confidence interval.

## Discussion

Despite efforts to stop early pregnancies, teenage pregnancy has been continued as a global public health problem. Therefore, this study was designed to examine the trend and pinpoint factors that contributed to the observed reduction in teenage pregnancy during the study period. The factors associated with teenage pregnancy have been the subject of previous investigations. To the best of our knowledge, there is limited evidence on the factors that contributed to the change in teenage pregnancy in Ethiopia.

The results of this study revealed that trends in teenage pregnancy prevalence decreased significantly between 2005 and 2011, even though it showed an increase between 2011 and 2016. From 2005 to 2011, the teenage pregnancy prevalence decreased by 4.3%, while it increased by 0.2% between 2011 and 2016, with an overall decrease of 4.1% from 2005 to 2016. This decline rate in the prevalence of teenage pregnancy has been consistent with Sustainable Development Goal target 3.7.2 and study findings on the trend of teenage pregnancy from 2000 to 2011 in Eastern Africa (3.5%)^27^, but higher than the study findings from West Africa (1.7%)^27^ and North America (1.2%)^28^. This could be due to the launching of the Health Extension Programme and improving access and utilization of maternal health services and decreasing the unmet need for family planning^29^. Increased political will, donor assistance, and non-governmental organization efforts to reduce early marriage in Ethiopia^30,31^ may also have contributed to this decline in teenage pregnancy prevalence.

Knowing the trend is important, but it's also important to understand what caused the reduction in teenage pregnancy and what factors are helping to reduce it to evaluate the current implementation strategies. The overall decomposition analysis revealed that the change in teenage pregnancy due to the difference in the effects of characteristics was not significant. Keeping coefficient changes constant, the disparity in the composition of women over time was significant and responsible for 49.8% of the decline in teenage pregnancy over the entire sample survey period (2005–2016).

Among the compositional factors, the effect of religion, educational status, and wealth index before the survey were significantly associated with the reduction in teenage pregnancy. An increase in the proportion of Muslim followers in the survey had a significant negative impact on the decline in teenage pregnancy. This finding was consistent with a study conducted in Malawi and Sri Lanka^32,33^. The possible justification might be Muslims tend to have an earlier marriage, and low use of family planning^34^ which is leading them to become extremely young mothers. However, further study is needed to investigate the effect of religion on teenage pregnancy.

An increase in the compositions of women who resides in rich households also showed a statistically significant positive contribution to the decline in teenage pregnancy. This finding was similar to a study conducted in Uganda^35^, Malawi^32^, Ethiopia^18,36^, South Sudan^37^, and sub-Saharan Africa^16,27^. A possible pathway of this influence could be that women with the lowest income tend to marry at an early age as girls’ families benefit from dowries (provided by the partner’s family often as cattle), while those with the highest income continue with their education and other career goals^38^.

The predominant decline in teenage pregnancy was due to the compositional change in teenagers’ primary or above education attainment. An increase in the proportion of teenagers having primary school education or above in the study period had a 66.4% contribution to the decline of teenage pregnancy, similar to what had been reported in other studies in Africa^16,20,32,35,37^. Teenagers who receive an education will have a greater understanding of sexual and reproductive health, including conception and fertility. The more education a girl has, the more likely she is informed about ways of preventing early pregnancy, and the more aware she is of contraceptive options. It has been also proposed that education increases adolescent prospects for success and acts as a deterrent to early parenthood^39–41^. Hence, to reduce the high rate of teenage pregnancy, governmental stewardship towards making primary and secondary school more accessible in Ethiopia is strongly important. Moreover, some cultural views that impede the education of girl children should be changed.

## Conclusion

Teenage pregnancy decreased significantly in the study period; however, it is still unacceptably high. The study has shown that changes in compositional factors of teenagers was responsible for the observed reduction in the prevalence of teen pregnancy rates in Ethiopia. Educational status, religion, and wealth index were found to be significant factors that contributed to the reduction in teenage pregnancy. Therefore, intervention programs targeting adolescents should address the socio-economic inequalities of these influential factors to reducing teenage pregnancy and related complications.

## Data Availability

The dataset is available from the DHS program official database https://www.measuredhs.com.

## Abbreviations

AOR: Adjusted odds ratio
CI: Confidence interval
DHS: Demographic and Health Survey
EAs: Enumeration areas
EDHS: Ethiopia Demographic and Health Survey
LMIC: Lower- and middle-income countries
SRH: Sexual and reproductive health
WHO: World Health Organization
